# Presence of anaplastic lymphoma kinase in inflammatory breast cancer

**DOI:** 10.1186/2193-1801-2-497

**Published:** 2013-10-01

**Authors:** Fredika M Robertson, Emanuel F Petricoin III, Steven J Van Laere, Francois Bertucci, Khoi Chu, Sandra V Fernandez, Zhaomei Mu, Katherine Alpaugh, Jianming Pei, Rita Circo, Julia Wulfkuhle, Zaiming Ye, Kimberly M Boley, Hui Liu, Ricardo Moraes, Xuejun Zhang, Ruggero DeMaria, Sanford H Barsky, Guoxian Sun, Massimo Cristofanilli

**Affiliations:** Department of Experimental Therapeutics, The University of Texas MD Anderson Cancer Center, Houston, TX 77030 USA; The Center for Applied Proteomics and Molecular Medicine, George Mason University, Manassas, VA USA; Department of Oncology, KU Leuven, Herestraat 49, Leuven, 3000 Belgium; Department of Molecular Oncology, Institut Paoli-Calmettes Marseille, Marseille, CEDEX 9, France; Department of Medical Oncology, Thomas Jefferson University, Philadelphia, PA 19107 USA; Protocol Support Laboratory, Fox Chase Cancer Center, Philadelphia, PA 19111 USA; Cancer Biology Laboratory, Fox Chase Cancer Center, Philadelphia, PA 19111 USA; Istituto Regina Elena, Rome, Italy; Department of Pathology, The University of Nevada School of Medicine, Reno, NV 89557 USA; Genzyme Genetics, New York, NY 10019 USA; Department of Experimental Therapeutics, The University of Texas MD Anderson Cancer Center, Unit 1950, 1901 East Road, South Campus Research Building 4; Office 3.1009, Houston, TX 77230-1429 USA

**Keywords:** Inflammatory breast cancer, Anaplastic lymphoma kinase, Reverse phase protein arrays, Crizotinib

## Abstract

Although Inflammatory Breast Cancer (IBC) is recognized as the most metastatic variant of locally advanced breast cancer, the molecular basis for the distinct clinical presentation and accelerated program of metastasis of IBC is unknown. Reverse phase protein arrays revealed activation of the receptor tyrosine kinase, anaplastic lymphoma kinase (ALK) and biochemically-linked downstream signaling molecules including JAK1/STAT3, AKT, mTor, PDK1, and AMPKβ in pre-clinical models of IBC. To evaluate the clinical relevance of ALK in IBC, analysis of 25 IBC patient tumors using the FDA approved diagnostic test for ALK genetic abnormalities was performed. These studies revealed that 20/25 (80%) had either increased ALK copy number, low level ALK gene amplification, or ALK gene expression, with a prevalence of ALK alterations in basal-like IBC. One of 25 patients was identified as having an EML4-ALK translocation. The generality of gains in ALK copy number in basal-like breast tumors with IBC characteristics was demonstrated by analysis of 479 breast tumors using the TGCA data-base and our newly developed 79 IBC-like gene signature. The small molecule dual tyrosine kinase cMET/ALK inhibitor, Crizotinib (PF-02341066/Xalkori®, Pfizer Inc), induced both cytotoxicity (IC_50_ = 0.89 μM) and apoptosis, with abrogation of pALK signaling in IBC tumor cells and in FC-IBC01 tumor xenograft model, a new IBC model derived from pleural effusion cells isolated from an ALK^+^ IBC patient. Based on these studies, IBC patients are currently being evaluated for the presence of ALK genetic abnormalities and when eligible, are being enrolled into clinical trials evaluating ALK targeted therapeutics.

**Electronic supplementary material:**

The online version of this article (doi:10.1186/2193-1801-2-497) contains supplementary material, which is available to authorized users.

## Introduction

Inflammatory breast cancer (IBC) is the most metastatic form of breast cancer (Robertson et al. 2010). IBC accounts for an estimated 24% of cases of advanced stage (Stage IIIb, IIIc or IV) breast cancers (Buzdar et al. 1995). Inflammatory breast cancer (IBC) has been defined as a clinical-pathologic entity characterized by diffuse erythema and edema (peau d’orange) involving a third or more of the skin of the breast (American Joint Committee on Cancer Staging Manual 2010). The swelling and enlargement of the breast and the appearance of dimpled skin defined as "peau d’ orange" is associated with the presence of tightly aggregated tumor cells, defined as tumor emboli, that have robust expression of E-cadherin and are encircled by dermal lymphatic vessels (Cristofanilli et al. 2007;Vermeulen et al. 2010). The involvement of the dermal lymphatics provides an avenue for rapid metastasis, associated with the common clinical and pathological signs of axillary and other loco-regional lymph node involvement in IBC patients at the time of first diagnosis (Robertson et al. 2010). Despite the development of multi-modality treatment strategies over the past 30 years that have increased overall survival of patients with non-IBC locally advanced breast cancers, there has been no significant change in survival of IBC patients during this same time period (Gonzalez-Angulo et al. 2007). The average survival of IBC patients (2.9 years) is significantly less than the survival rate of patients diagnosed with non-IBC locally advanced breast cancer (6.4 years) and the >10-year survival rate of patients with non-T4 (i.e., non-locally advanced) breast cancer (Hance et al. 2005).

Only a few genes, such as Rho C GTPase, have been associated with the invasive phenotype of IBC (Van Golen et al. 2000;Kleer et al. 2005) and the underlying genetic changes in IBC remain largely undefined. The lack of the understanding of the molecular underpinnings of IBC points out the critical need to identify abnormalities in gene and protein signaling pathways that are activated in IBC in order to provide new therapeutic strategies to IBC patients who are routinely excluded from clinical trials.

The present studies present first time evidence for the activation of anaplastic lymphoma kinase (ALK) pathway activation in pre-clinical models of IBC, that was consistent with detection of increased gains in copy numbers of ALK, low level ALK gene amplification, ALK gene expression or more rarely, the presence of EML4-ALK translocation in IBC breast tumors. Analysis of breast tumors in the TGCA database revealed a significant association between basal-like breast tumors that have characteristics of IBC breast tumors and gains in ALK copy number. The dual cMET/ALK inhibitor, Crizotinib, induced significant cytotoxicity in ALK + IBC cell lines and *in vivo* studies revealed that this agent induced significant apoptosis in ALK^+^ IBC xenografts which was associated with inhibition of phospho-ALK signaling activation. Collectively, these results suggest that ALK serves as a therapeutic target for IBC and indicate that strategies targeting ALK should be considered for evaluation in clinical trials.

## Materials and methods

### Cell lines

The SUM149, SUM159 and SUM190 cell lines were purchased from Asterand (Detroit, MI). The MDA-IBC3 cells were obtained from W.A. Woodward and KPL-4 cells were obtained from N. T. Ueno, The University of Texas MD Anderson Cancer Center. All other cell lines, AU565, MDA-MB-231, MDA-MB-468, MCF-7, and SKBR3, were purchased from American Type Culture Collection (ATCC;Manassas, VA). The new models of ALK^+^ IBC, designated as FC-IBC01 and FC-IBC02, were developed in the laboratories of FM Robertson, The University of Texas MD Anderson and M Cristofanilli, Thomas Jefferson University, using tumor cells freshly isolated from IBC patients with disease progression as evidenced by pleural effusion. Pleural fluids were removed by thoracentesis using an IRB approved protocol, with patient consent; tumor cells were isolated and served as the source to derive new IBC cell lines and xenograft models (Fernandez et al. 2013). Mary-X is a stable transplantable IBC xenograft derived from a patient with primary IBC and developed by Sanford H. Barsky (Alpaugh et al. 1999). Identity of all cell lines was validated based on STR analysis performed by the MD Anderson Cell Analysis core laboratory.

### Reverse phase protein microarray analysis

Pathway activation mapping was performed by reverse phase protein microarray (RPMA) as previously described (Paweletz et al. 2001;Wulfkuhle et al. 2008;Einspahr et al. 2012;Sheehan et al. 2005). Protein signaling analytes were chosen for analysis based on their involvement in key aspects of tumorigenesis: growth, survival, autophagy, apoptosis, differentiation, adhesion, motility, and inflammation. All antibodies were validated for single band specificity as well as for ligand-induction (for phospho-specific antibodies) by Western Blotting. Continuous variable RPMA data generated were subjected to both unsupervised and supervised statistical analysis. Statistical analyses were performed on final RPMA intensity values obtained using SAS version 9 software or JMP v5.0 (SAS Institute, Cary, NC). Initially, the distribution of variables was checked. If the distribution of variables for the analyzed groups (e.g. IBC v non-IBC) was normal, a two-sample t-test was performed. If the variances of two groups were equal, two-sample t-test with a pooled variance procedure was used to compare the means of intensity between two groups. Otherwise, two-sample t-test without a pooled variance procedure was adopted. For non-normally distributed variables, the Wilcoxon rank sum test was used. All significance levels were set at p ≤ 0.05.

### Analysis of ALK genetic abnormalities

Methods for FISH analysis of ALK genetic abnormalities were as previously published (Shaw et al. 2011). Results of the FISH analysis were read by Dr. Guoxian Sun, a board certified pathologist in the Genzyme Genetics CLIA approved diagnostic laboratory. Results were independently validated by direct PCR and CMA analysis. Fluorescence microscopy images were taken of each tumor specimen to quantitate the heterogeneity of ALK copy number and to assess the location of the FISH probes.

### Chromosomal analysis

Affymetrix CytoScan™ HD arrays were used to evaluate copy number and loss of heterozygosity (LOH) in samples of IBC and non-IBC breast cancer cell lines. These arrays contain more than 2.6 million copy number markers of which 750,000 are "genotype–able" SNPs and 1.9 million are non–polymorphic probes. DNA was isolated using Gentra Puregene Cell kit (Qiagen) based on manufacturers protocols. Copy number and genotyping analyses were performed using Affymetrix Chromosome Analysis Suite (ChAS) software.

### Analysis of ALK gene expression and ALK amplification in TCGA samples classified as "IBC-like" and non-IBC-like (nIBC)

We recently reported the development of a nearest shrunken centroid classification model based on the expression of 79 IBC-specific and molecular subtype-independent genes that was able to correctly discriminate between samples from patients with and without IBC (Van Laere et al. 2013). Using this model, we analyzed a series of 479 samples from patients with non-IBC breast cancer (nIBC) for which gene expression data were available through the TCGA project (Cancer Genome Atlas Network 2012). Based on the 79-gene signature that we developed, tumor samples were classified as either having "IBC-like" or "nIBC-like" characteristics. Prior to the application of the model, TCGA expression data were normalized using regression models to obtain a data distribution comparable to the data distribution of the training set on which the nearest shrunken centroid algorithm has been trained. To classify the same samples according to the molecular subtypes, the PAM50 algorithm (Parker et al. 2009) was applied. Finally, putative ALK copy number alterations (CNAs), estimated using GISTIC 2.0 were retrieved and were categorized as follows: -2 = homozygous deletion; -1 = hemizygous deletion; 0 = neutral/no change; 1 = gain; 2 = high-level amplification. All data (i.e. mRNA expression data and ALK CNA data) were retrieved from the World Wide Web (http://www.cbioportal.org).

### Microarray analysis of breast tumor cell lines

Cells were isolated and total RNA was extracted using RNeasy kits (Qiagen; Gaithersburg, MD), with RNA integrity determined using an Agilent Bioanalyzer 2100 (Agilent Technologies, Inc; Santa Clara, CA. Whole transcriptome analysis of cells was performed using Genechip® U133 p v2 microarray platform (Affymetrix, Inc; Santa Clara CA) in the RNA core laboratory at the University of Texas MD Anderson Cancer Center. Microarrays were scanned using a GeneChip Scanner 7G (Affymetrix, Santa Clara, CA), Microarray date files were imported using dChip v.1.3 software, Nexus and IPA algorithms, data was normalized using invariant set normalization and analyzed to detect significant differences in gene expression. The output is a log2-transformed expression index data of each probe set. Differences between the expression of genes of interest between IBC cell lines and non-IBC cell lines were analyzed and are represented as a heatmap.

### Analysis of cytotoxicity of Crizotinib in cell lines

Cell proliferation was assayed using the ProMega CellTiter Cell Proliferation Assay (Promega, Fitchburg, WI) based on manufacturers protocols. MDA-MB-231, SUM159, and SUM149 cells were seeded into a 96 well plate at 1500 cells per well and H2228, MCF-7, SUM190, MDA-IBC-3, and freshly isolated tumor cells from the patient designated as FC-IBC01 were seeded at 4000 cells/well, allowed to attach overnight and treated with Crizotinib (Selleck Chemical, Houston, TX) dissolved in DMSO at the indicated concentrations. Experiments were terminated at 72 hrs following treatment, processed according to the manufacturer’s instructions and plates were read at 490 nm using a BioTek plate reader (Winooski, VT). Data analysis was performed using Prism GraphPad 5.0 (GraphPad Software). Studies were performed at least three times with similar results.

### Xenograft implantation

All experiments involving animals were conducted in accordance with protocols approved by the University of Texas MD Anderson Cancer Center Institutional Animal Care and Use Committee (IACUC). FC-IBC01 cells and Mary-X cells (5 × 10^5^) were injected either into the lower left mammary fat pad or subcutaneously into the hind flanks of female 6–8 week old NOD.Cg-Prkdc^scid^ Il2rg^tm1Wjl^/SzJ mice to evaluate primary tumor growth and for the presence of tumor emboli formation, respectively. Mice bearing tumors of at least 200 mm^3^ were treated either with 83 mg/kg Crizotinib or DMSO vehicle control once daily for three days via gavage and sacrificed at 24 hr following the last dose.

### Immunohistochemistry and microscopy

FC-IBC01 and Mary-X tumors and associated skin in the case of hind flank injection studies were collected at necropsy and fixed in 10% neutral buffered formalin. Tissues were paraffin-embedded, sectioned, and stained with H&E. Immunochemical methods used to stain tissues for phospho-proteins, E-cadherin and podoplanin are as previously reported (Robertson et al. 2012). Antibodies used included p-ALK Y-1586 antibody (Abnova, Walnut Creek, CA) p-AKT-Ser-473 (Cell Signaling, Danvers, MA) and p-mTOR-Ser-2448 (Cell Signaling). The DNA dye, TOPRO-3 (Invitrogen/Life Technologies) was used to identify nuclear DNA. For negative controls, tissue was processed in the same way with the omission of primary antibody. Images were examined and captured using a LSM 510 confocal laser scanning system (Carl Zeiss, Thornwood, NY) at 25× magnification.

### TUNEL staining and quantitation

Apoptosis was detected using Roche fluorescence labeled *In situ* cell death detection kit, (Roche Applied Science, Hague, IN), based on manufacturers protocols. Positive controls tissues for the TUNEL assay were DMSO treated tissues treated with recombinant DNase I and negative controls were tissues stained reaction mixture lacking terminal transferase. To quantitate TUNEL staining, three tumors from separate mice were used for each of the control and drug treated groups). Images for TUNEL stained cells and TOPRO-3 labeled nuclei were captured on five randomly chosen fields for each section. Image J software (http://rsbweb.nih.gov/ij/) was used to count the number of stained cells and nuclei. The comparison between vehicle control and Crizotinib treated groups were performed to determine the significance between groups using Student two-tailed T-test. Quantitation of Crizotinib-induced changes in staining of p-Akt-Ser473 and p-mTor-Ser2448 were performed using methods described above.

### Western blot analysis of cMET and phospho-cMET

The antibodies directed against total cMET, alpha chain of phospho-Y1234/1235 cMET and beta chain of phospho-Y1234/1235 cMET were purchased from Cell Signaling (Danvers, MA). Protein was normalized to GAPDH, used as a loading control. Cells were isolated to obtain cell pellets, which were dried and then lysed in 1% M-PER lysis buffer (Thermo Fisher, Rockford, IL). Protein concentrations of lysates were determined using a total protein assay (Bio-Rad Laboratories, Hercules, CA). Equal amounts of protein (100 μg) were loaded and then separated using 10% polyacrylamide gels (Bio-Rad). Proteins were transferred to nitrocellulose membranes (GE Healthcare, Piscataway, NJ), blocked for non-specific binding using a buffer containing 1X PBS, 0.1% Tween-20, and 5% milk and then probed with 1:1000 dilution of cMET, phospho-cMET antibodies followed by incubation with horseradish peroxidase-conjugated secondary antibodies (GE Healthcare). Protein bands were visualized using Chemiglow enhanced chemiluminescence system (Alpha Imager, San Leandro, CA) and densitometric analysis was used to quantitate changes in proteins. Experiments were repeated three times and representative Western blots are shown.

## Results

### Functional protein pathway of breast cancer cell lines

**Figure 1:**
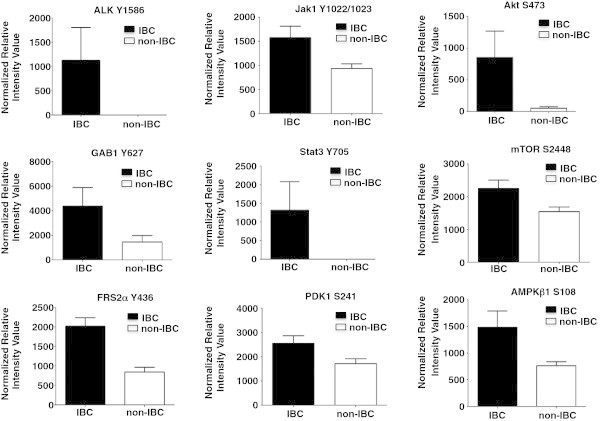
**Activation of ALK signaling network in pre-clinical models of IBC.** Reverse Phase Protein Microarray analysis of human IBC cell lines and non-IBC human breast tumor cell lines that were cultured under low adherence conditions supporting self-renewal revealed activation of multiple members of the receptor tyrosine kinase (RTK) ALK signaling network, including JAK1/STAT3, AKT, mTor, PDK-1 and AMP kinase β. The *P* values for the level of phosphorylation of these signaling molecules in IBC cell lines compared with non-IBC cell lines was set at p ≤ 0.05 and the individual *P* values are described in the Results section.

Reverse Phase Protein Microarray (RPMA), which is a powerful pathway activation mapping technology that we previously developed and described (Paweletz et al. 2001;Wulfkuhle et al. 2008;Einspahr et al. 2012;Sheehan et al. 2005), was utilized to map 150 key signaling proteins in human IBC cell lines (Mary-X, MDA-IBC-3, SUM 190, and SUM 149) and non-IBC human breast cancer cell lines (MCF7, MDA-MB-468, SUM159, and MDA-MB-231). RPMA analysis revealed that IBC cell lines exhibited activation of multiple members of the ALK receptor tyrosine kinase (RTK) signaling network (Figure 1). This activation included phosphorylation of ALK itself at the tyrosine (Y) 1586 phosphorylation site, p ≤ 0.05), and activation of the RTK docking proteins, GAB1 ((Y627), p ≤ 0.02) and FRS2 alpha (Y436, p ≤ 0.02). In addition, IBC cell lines exhibited activation of RTK-driven JAK-STAT signaling (JAK1 (Y1022/1023, p ≤ 0.04), STAT3 (Y705, p ≤ 0.05), as well as the RTK-driven AKT-mTOR signaling pathways, including PDK1 (Serine (S) 241), p ≤ 0.03); AKT (S473, p ≤ 0.004 and Threonine (T) 308 p ≤ 0.005); mTOR (S2448, p ≤ 0.02), and AMPK Beta (S108, p ≤ 0.05). In contrast, the non-IBC cell lines including MCF-7, MDA-MB-231, SUM159 and MDA-MB-468 had no ALK phosphorylation above array background.

### ALK in IBC patient tumors

**Figure 2:**
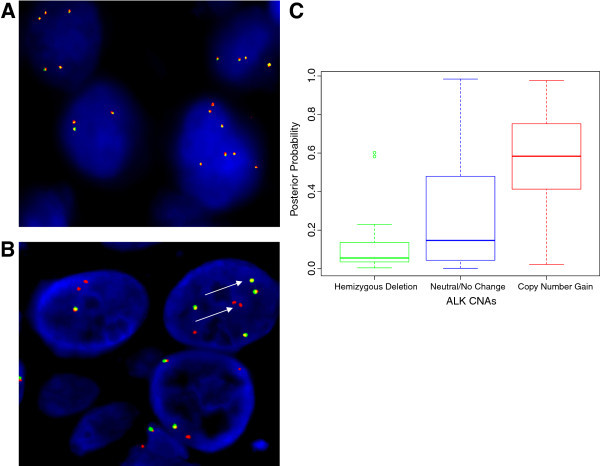
**FISH analysis of ALK genetic abnormalities in IBC patient tumors and Levels of ALK Gene Expression in Breast Tumors. A**. Immunofluorescence image of FISH analysis of IBC tumor showing heterogeneity of ALK copy number, varying from 3–8 copies of ALK. **B**. Immunofluorescence image of FISH analysis of IBC tumor showing one of the two fusion signals separated as one red and one green signal in 76% of nuclei scored, consistent with the presence of EML4-ALK translocation (arrows). **C**. The posterior probabilities of samples to be classified as "IBC-like" in function associated with ALK copy number alterations (CNAs) are shown in boxplot-format. All observed differences were statistically significant (*P* ≤ 0.010). There was a positive and significant association between breast tumor samples classified as basal-like with IBC characteristics and ALK copy number alterations.

A set of 25 randomly selected IBC patient tumors were analyzed using the FDA approved fluorescence in situ hybridization (FISH) detection method based on the Vysis ALK Break Apart Probe for detection of EML-4-ALK translocation and gene amplification (Shaw et al. 2011). These studies were performed independently and reviewed by a board certified pathologist at a CLIA-approved Genzyme Genetics Laboratory, Dr. Guoxian Sun. As shown in Table 1, 20/25 IBC patient tumor samples had some type of ALK genetic aberrations including ALK copy numbers, ALK gene amplification and in the case of 1 IBC patient, EML4-ALK translocation. As an example of the interpretation of FISH analysis for one IBC sample reflective of the heterogeneity of ALK copy number alterations or ALK amplification detected in IBC patients, the report was "negative for rearrangements involving the ALK gene, with three to four copies of ALK observed in 59.0% of cells, five to six copies of ALK observed in 11.0% of cells and seven to eight copies of ALK were observed in 6.0% of cells, suggesting the presence of a neoplasm with gains of chromosome 2 or 2p". Figure 2A is a two-color immunofluorescence image of the FISH analysis for this specific IBC sample. When an ALK rearrangement is present in a tumor, whether it is an inversion or translocation, one of the two fusion signals separates as one red and one green signal (Figure 2B, arrows). As shown in Figure 2B, separated signals were present in 1 IBC tumor that had EML-4-ALK genetic abnormality in 76% of nuclei scored, which is outside the normal limits. The patterns observed suggest the presence of a concomitant deletion of the 5′ centromeric green probe signal, which is a common finding in NSCLC.

**Table 1:** **ALK genetic abnormalities in IBC patient tumors**

Subtype of IBC patients	No. patients with ALK abnormalities	Percentage of cells with increase ALK copy number
ER/PR/Her2 negative	13[1 with EML4-ALK]	16-75%
HR+/Her2 negative	4	9-65%
Her2+	3	33-51%

### Analysis of ALK amplification in TCGA-samples classified as "IBC-like" and "nIBC-like"

Using the 79-gene signature model that we recently developed (Van Laere et al. 2013), approximately 25% of TCGA breast tumor samples were classified as "IBC-like". Samples classified as "IBC-like" were significantly more often of the basal-like subtype (65% *vs.* 10% for samples classified as "nIBC-like"; P < 0.001). Data on ALK copy number alterations (CNAs) were available for 455/479 samples of the TGCA database. Hemizygous deletions of ALK (GISTIC class -1) were reported in 32 samples (7%) and a copy number gain (GISTIC class +1) was reported in 43 samples (9%). In 380 samples (84%), no CNAs were detected for ALK. Sixty percent of the samples with an ALK copy number gain were classified as "IBC-like" whereas 93% of the samples with a ALK hemizygous deletion were classified as "nIBC-like" (Chi-square test; P < 0.001). The posterior probabilities of samples to be classified as "IBC-like" associated with the extent of ALK CNAs are provided in boxplot-format in Figure 2C. All observed differences were statistically significant (all *P* < 0.010). Evaluation of the molecular subtypes in relationship with ALK CNAs revealed that 72% of the ALK copy number gains were present in samples classified as basal-like. In contrast, 80% of the hemizygous ALK deletions were observed in samples classified as luminal A or luminal B subtypes. Of note, 98% of the "normal-like" samples classified as ALK copy number neutral. Given these association, we sought to determine the influence of the molecular subtypes on the association between ALK CNAs and the "IBC-like"/"nIBC-like"-classification. Multivariate regression analysis revealed that the "IBC-like"/"nIBC-like" classification was associated with ALK CNAs, independent of the molecular subtypes (OR = 1.106; 95%C.I. = 1.001-1.222; P = 0.048). These data suggest that, at least in non-IBC breast tumors, the presence of ALK copy number alterations were more likely to occur in those tumors that had characteristics gene expression profiles similar to those from patients with IBC.

### Development of ALK + IBC pre-clinical models

Since there are few pre-clinical IBC models available to study the effects of the small molecule cMET/ALK inhibitor Crizotinib, we developed an ALK^+^ pre-clinical model of IBC using tumor cells freshly isolated from IBC patient with disease progression evidenced by pleural effusion. Tumor cells were isolated from pleural effusion of a 48 year old woman with stage IIIC triple negative IBC at time of initial diagnosis who had received neoadjuvant chemotherapy including Cytoxan, Adriamycin + Taxane, carboplatin and gemcitabine, with preoperative radiotherapy. She had extensive residual disease in the breast and local lymph nodes, suggesting resistant disease. She developed progressive disease a few weeks following surgery, with symptomatic pleural effusion. Bilateral pleural effusions were visible in the right quadrant. Pleural fluid was removed by thoracentesis using an IRB approved protocol, with patient consent, and these tumor cells, which we designated as FC-IBC01, were isolated. The freshly isolated FC-IBC01 tumor cells served as the source of cells to analyze the effects of Crizotinib and to derive a new IBC cell line and xenograft model used for to assess ALK gene expression, and in vivo response to Crizotinib.

### ALK in IBC cell lines and xenograft models

**Figure 3:**
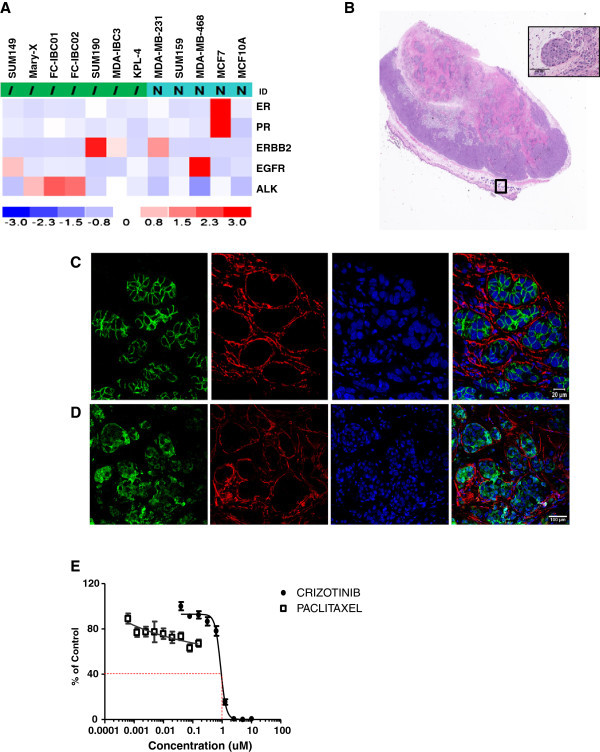
**ALK Gene Expression of Pre-Clinical Models of IBC. A**. Analysis of gene expression levels of ER/PR/Her2, EGFR and ALK in each of the 7 available pre-clinical models of IBC including SUM149, Mary-X, FC-IBC01, FC-IBC02, SUM190, MDA-IBC-3 and KPL-4 cells and non-IBC human breast tumor cell lines MDA-MB-231, SUM159 and MCF-7. The highest levels of ALK gene expression was detected in the triple negative IBC cell lines FC-IBC01, FC-IBC02 and Mary-X, which each recapitulate the formation of IBC tumor emboli *in vivo*. **B**. Light micrograph of histology of FC-IBC01 xenograft showing poorly differentiated tumor with high nuclear grade and prominent mitotic activity, with visible invasion through the hypodermis into the dermal-epidermal junction. Inset: Distinct tumor emboli are visible within the dermal layer of the skin in the H&E section of FC-IBC01 xenograft tissue (inset). **C**. Confocal microscopy combined with triple color immunofluorescence staining demonstrates that mice bearing FC-IBC01 tumors form tumor emboli within the dermis that express E-cadherin (green fluorescence) and are enwrapped by lymphatic vessels, identified by specific staining for podoplanin (red fluorescence). The DNA dye Topro-3 (blue fluorescence) identifies nuclei. **D**. FC-IBC01 tumor emboli contain ALK protein (green fluorescence) and are encircled by podoplanin stained lymphatic endothelium (red fluorescence). **E**. Dose response analysis of tumors cells freshly isolated from the patient designated as FC-IBC01 demonstrating response to Crizotinib and resistance to Paclitaxel.

Of the 7 IBC cell lines examined, the newly developed cell lines and pre-clinical models of IBC designated as FC-IBC01 and FC-IBC02, in addition to the Mary-X cells, which all classify within the basal like subtype and form tumor emboli when injected *in vivo*, expressed the highest levels of ALK gene expression (Figure 3A).

Additional file 1: Table S1 shows results of Chromosomal Microarray Analysis (CMA) of all IBC cell lines, revealing that there are a number of ALK genetic abnormalities in pre-clinical models of IBC, including increased copy number, gene amplification (ALK > 3) and in the case of FC-IBC01 uniparental disomy. This analysis also demonstrated that focal adhesion kinase (FAK) and the stem cell marker CD44 may also be likely therapeutic targets in IBC based on their levels of amplification in the pre-clinical models of IBC that recapitulate the formation of tumor emboli.

FC-IBC01 tumor cells were injected subcutaneously into the right hind flanks of NOD.Cg-Prkdcscid Il2rgtm1Wjl/SzJ mice, and poorly differentiated tumors with high nuclear grade and prominent mitotic activity developed within 45 days, with visible invasion through the hypodermis into the dermal-epidermal junction (Figure 3A). Numerous tumor emboli were visible within the dermis adjacent to the primary FC-IBC01 xenograft (Figure 3A, inset) which were found to have robust expression of E-cadherin (green fluorescence) (Figure 3C), which is characteristic of the skin involvement of this variant of breast cancer that is commonly observed in IBC patients. The FC-IBC01 tumor emboli that expressed E-cadherin were enwrapped by lymphatic vessels, which are identified by specific staining for podoplanin (red fluorescence) (Figure 3C). The FC-IBC01 tumor emboli, which were encircled by lymphatic endothelium (red fluorescence), also expressed ALK protein (green fluorescence) (Figure 3D). Nuclear DNA is stained with the DNA dye TOPRO-3 (blue fluorescence).

### IBC tumor cells are sensitive to the small molecule ALK inhibitor, Crizotinib

**Figure 4:**
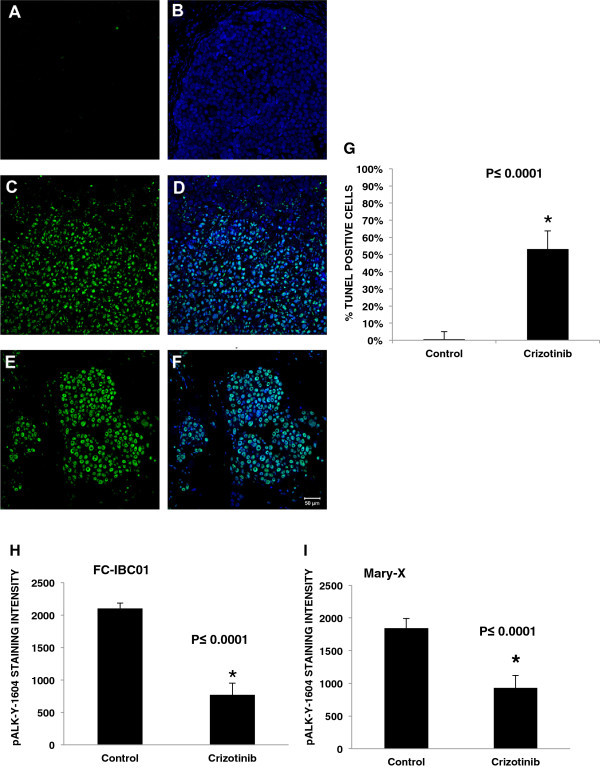
**Effects of Crizotinib in Pre-clinical Models of ALK + IBC. A-B**. Treatment of mice bearing FC-IBC01 xenografts with DMSO vehicle control had no detectable apoptosis as determined by detection of TUNEL staining as a marker of programmed cell death. Figure 4 A shows the lack of green fluorescence associated with TUNEL staining and Figure 4 B shows a lack of green fluorescence which detects TUNEL staining, with detection of blue fluorescence which is associated with nuclear DNA based on Topro-3 staining. **C** and **D**. Treatment of mice bearing FC-IBC01 xenografts with 83 mg/kg Crizotinib resulted in significant apoptosis of FC-IBC01 tumor cells, as assessed by detection of TUNEL as a marker for programmed cell death, as denoted by the green fluorescence (Figure 4 C) and double label of green fluorescence which detects TUNEL staining and blue fluorescence detects nuclear DNA based on Topro-3 staining (Figure 4 D). **E** and **F**. Positive control for TUNEL staining using DNAse I treatment of xenograft tissues. **G**. Comparative quantitative analysis of Crizotinib-induced apoptosis in FC-IBC01 tumor xenografts demonstrates a significant increase in detection of TUNEL positive cells (*P* ≤ 0.0001). **H** and **I**. Comparative analysis of inhibitory effects of Crizotinib on phospho-ALK-Y-1604 signaling activation in FC-IBC01 and Mary-X pre-clinical models of IBC. Treatment of mice bearing FC-IBC01 (H) or Mary-X (I) with 83 mg/kg Crizotinib significantly inhibited phospho-ALK- Y-1604 (*P* ≤ 0.0001).

The dose response of freshly isolated FC-IBC01 cells to the small molecule ALK inhibitor, Crizotinib, is shown in Figure 3E. Crizotinib was cytotoxic against FC-IBC01 cells, with an IC_50_ of 0.89 μM (Figure 3E; Table 2). SUM149 cells, which we have found to express phospho-cMET protein (Additional file 2: Figure S1), were also responsive to the cytotoxic effects of the dual cMET/ALK inhibitor, Crizotinib. The range of IC_50_ doses for the IBC cell lines that express either ALK or cMET mRNA is consistent with the IC_50_ concentration of Crizotinib in the H2888 NSCLC cell line, which has an EML4-ALK translocation, and for the IMR-32 neuroblastoma cell line, IMR-32 (Table 2) which harbors full length wild type oncogenic ALK. Studies were performed to evaluate the effects of treatment of mice bearing FC-IBC01 xenografts with Crizotinib. Treatment of tumor bearing mice with daily doses of 83 mg/kg Crizotinib administered via gavage induced significant apoptosis of FC-IBC01 tumor cells, detected by TUNEL staining as the marker for programmed cell death (Figure 4A-G). The TUNEL staining appears as green fluorescence and the nuclear DNA is stained with the DNA dye TOPRO-3. Figure 4A and B shows the lack of TUNEL staining in FC-IBC01 xenograft tissue isolated from mice treated with the DMSO vehicle control. Figure 4C and D shows the representative increase in TUNEL staining in FC-IBC-01 xenograft tissue isolated from Crizotinib-treated mice. The positive control for TUNEL staining is shown in Figures 4E and F. Quantitation of the differences in TUNEL staining between vehicle control and Crizotinib-treated tissues demonstrates that this agent induced significant levels (P ≤ 0.0001) of apoptosis (Figure 4G). In addition to the significant apoptotic response, quantitative image analysis also revealed that Crizotinib significantly inhibited phospho-ALK-Y-1604 staining in both the FC-IBC01 and Mary-X models of IBC (Figure 4H and I; P ≤ 0.0001). Similarly, quantitative analysis of the effects of Crizotinib in xenograft tissues from mice bearing either FC-IBC01 or Mary-X tumors demonstrated that this cMET/ALK inhibitor also significantly diminished phospho-AKT-serine 473 and phospho-mTOR ser-2448 signaling activation (data not shown).

**Table 2:** **IC**_**50**_ **concentrations of Crizotinib**

Cell line	Crizotinib IC_50_
FC-IBC01	0.89 μM
Mary-X	0.87 μM
SUM149	0.77 μM
MDA-IBC-3	1.98 μM
SUM190	5.20 μM
KPL-4	6.45 μM
H2228 NSCLC EML4-ALK	0.834 μM
IMR-32 wt ALK	0.74 μM

## Discussion

The ALK receptor tyrosine kinase was initially identified as a member of the insulin receptor subfamily that acquires transforming capability when it is truncated and fused to NPM (nucleophosmin) in a chromosomal rearrangement that is common in anaplastic large cell lymphomas (ALCL) and in non-Hodgkin’s lymphoma with a T cell phenotype (Morris et al. 1994;Wellmann et al. 1995). Recent focus on ALK as a therapeutic target occurred due to the discovery of a fusion of ALK with echinoderm microtubule associated protein 4 (EML4-ALK) in a population of NSCLC patients who were highly responsive to the small molecule cMet/ALK inhibitor, Crizotinib (Xalkori; Pfizer, Inc, Groton, CT) (Pillai and Ramalingam 2012;Kwak et al. 2010;Kim et al. 2012;Tiseo et al. 2011). The clinical efficacy of Crizotinib in this patient population during early phase clinical trials paved the way for accelerated FDA approval of this targeted therapeutic (8/2011), in tandem with development and FDA approval of a diagnostic test that detects both EML4-ALK translocation and ALK copy number, and is used to select patients for enrollment into clinical trials with Crizotinib (Shaw et al. 2011). Recent reports from the results of the PROFILE study document the superiority of Crizotinib treatment in NSCLC patients with ALK genetic abnormalities compared with standard second line chemotherapy (Shaw et al. 2013;Pilotto et al. 2013). This clinical trial demonstrates the potential utility of early use of targeted therapeutics. Multiple other tumor types from a wide variety of organ sites have now been found to have different ALK abnormalities, other than NPM-ALK and EML4-ALK fusions, including increased ALK copy number, ALK amplification, ALK gene expression, missense point mutations, fusions between ALK and multiple genes and/or ALK signaling pathway activation (Kelleher and McDermott 2010). It is now clear that genetic abnormalities of ALK and ALK signal pathway activation are present in numerous tumor types, with other ALK abnormalities still to be discovered. The diversity of tumor types with a wide variety of ALK genetic abnormalities as well as ALK gene expression and activation of the ALK signaling pathway has prompted the suggestion that a new classification of "Alkomas" be used to denote tumors that have ALK as an oncogenic driver, regardless of their cell of origin (Mano 2012).

In contrast to studies identifying genetic abnormalities of ALK in other tumor types, results of investigations evaluating breast tumors for ALK genetic abnormalities have been inconsistent. While one study reported that 2.4% of breast tumors had translocation of EML4-ALK (Fukuyoshi et al. 2008), another study failed to detect the EML4-ALK fusion gene in breast tumors (Lin et al. 2009). Most recently, Lehmann et al (Lehmann et al. 2011) identified ALK as a signaling pathway important in triple negative breast cancers (TNBCs) and TNBC cell lines that had characteristics of mesenchymal cells and mesenchymal stem cells. Collectively, these observations suggest that EML4-ALK abnormalities are likely relatively rare in breast cancers in general, with ALK gene expression and activation of the ALK signaling pathway more common in TNBC. This observation has important implications, given that IBC patients typically have tumors that are either of the TNBC subtype or alternatively are Her-2^+^ (Van Laere et al. 2013). The observation of ALK gene expression in TNBC in general is consistent with the present results demonstrating the prevalence of increased ALK copy number, low level gene amplification and/or ALK pathway activation in IBC pre-clinical models of triple negative IBC; This observation is also consistent with the detection of ALK abnormalities in IBC tumors and with the identification of ALK copy gains in basal-like breast cancers that have an "IBC-like" gene signature.

Prior to the present studies, few genetic abnormalities or dysregulated signaling pathways had been identified in IBC. Using a functional protein pathway activation mapping approach coupled with genomic analysis approach, the present studies are the first to identify ALK signaling as a potential driver in pre-clinical models of IBC that recapitulate the formation of tumor emboli when grown as xenografts which we demonstrate have either ALK signaling activation, low level gene amplification, and/or ALK gene expression. These results suggest that IBC is characterized as having multiple changes in ALK that can occur at the gene level or at the protein pathway activation level. Based on these results, IBC patients are currently being screened for ALK genetic abnormalities and if eligible, have the opportunity to participate in clinical trials with ALK inhibitors [http://clinicaltrials.gov/show/NCT01283516]. Collectively, these studies represent an example of precision medicine focused on translating pre-clinical observations to benefit patients with this unique and lethal form of breast cancer.

## Electronic supplementary material

Additional file 1: Table S1: Chromosomal Microarray Analysis of Pre-Clinical Models of IBC. (PDF 81 KB)

Additional file 2: Figure S1: Comparative western blot analysis of total cMET protein and the 45 kD alpha chain and 145 kD beta chain forms of phospho-tyrosine (Y 1234/1235) cMET in 13 human breast tumor cell lines. The mature 45 KD form of cMET was detected primarily in SUM149 IBC cells as well as in AU565, MDA-MB-231, SUM159 and MDA-MB-468 breast tumor cells. The 145 kD beta chain form of phospho-Y 1234/1235 cMET was present in SUM149 cells, in SUM190, MDA-IBC3 and KPL-4 IBC cells and in AU565, MDA-MB 231, SUM159, MDA-MB-468 and in SKBR3 human breast tumor cells. In contrast, the 45 kD alpha chain form of phospho- Y 1234/1235 cMET was generally produced at low levels and detected in SUM149 IBC cells as well as in SUM159 breast tumor cells. (PDF 43 KB)

## Abbreviations

ALK: Anaplastic lymphoma kinase
CNAs: Copy number alterations
EML4: Echinoderm microtubule associated protein-like 4
FISH: Fluorescence in situ hybridization
FDA: Food and drug administration
IBC: Inflammatory breast cancer
n-IBC: Non-IBC
NSCLC: Non-small cell lung cancer
RTK: Receptor tyrosine kinase
RPMA: Reverse phase microarray
ser: Serine
TNBC: Triple negative breast cancer
Y: Tyrosine.
