# Supplementary material for: Presence of anaplastic lymphoma kinase in inflammatory breast cancer
Source: Springerplus. 2013 Oct 1;2:497. doi: 10.1186/2193-1801-2-497 (PMC3791224; doi:10.1186/2193-1801-2-497)
Supplement: Supplementary file 1 — Additional file 1: Table S1: Chromosomal Microarray Analysis of Pre-Clinical Models of IBC. (PDF 81 KB) [file 40064_2013_554_MOESM1_ESM.pdf]

Supplemental Table 1.
Chromosomal Microarray Analysis of Pre-Clinical Models of IBC

Gene and Location	Gene description	Copy number									
		IBC							Non-IBC		
		Triple negative				ER(-)PR(-)ErbB2+			ER(+)	Triple negative	
		FC-IBC01	FC-IBC02	Mary-X	SUM-149	MDA-IBC3	SUM-190	KPL4	MCF7	MDA-MB-231	SUM-159
**ALK** **2p23.2**	**Anaplastic lymphoma kinase, receptor tyrosine kinase, oncogene**	UPD	3.5-4	3	1.5	3	1	3	2	mos UPD	2
**EGFR** **7p12**	**Epidermal Growth Factor Receptor**	mos UPD	2.5-3	2.5	2.5-3	1	4	3	UPD	mos UPD	UPD
PTK2/FAK1 8q24.3	**Focal adhesion kinase 1**	4	6 HMZ	6 HMZ	2.5	2.5	2.5	4 hmz	2.5	2.5 hmz	2
**CD44** **11p13**	**CD44**	10 HMZ	8	UPD	3	1	1	2	1	2	2

Table 1. Copy number of some amplified genes in IBC and non IBC cells. Abbreviations: Hmz, homozygous; UPD, uniparental disomy (two copies with LOH); mos: mosaic.
