# Supplementary figures and images for: Presence of anaplastic lymphoma kinase in inflammatory breast cancer
Source: Springerplus. 2013 Oct 1;2:497. doi: 10.1186/2193-1801-2-497 (PMC3791224; doi:10.1186/2193-1801-2-497)

## Supplemental Figure 1

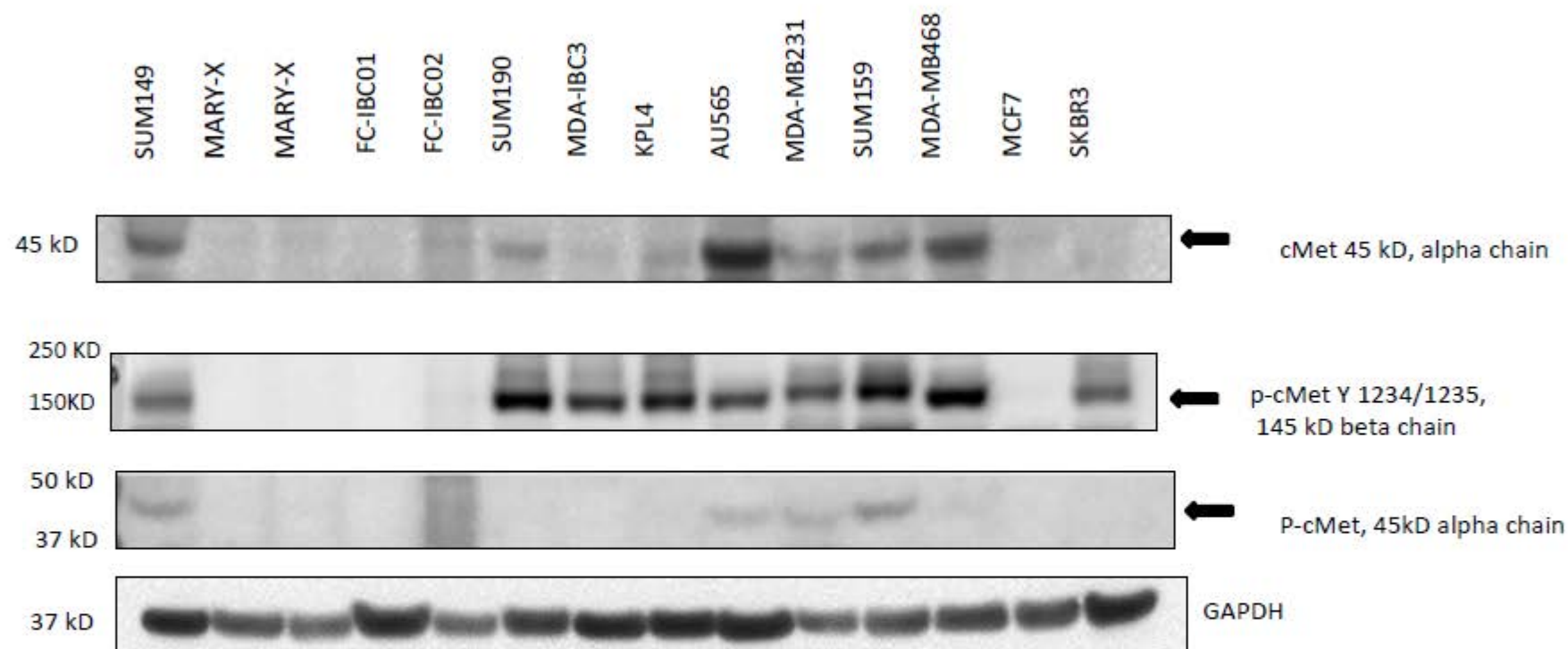

Supplement: Supplementary file 2 — Additional file 2: Figure S1: Comparative western blot analysis of total cMET protein and the 45 kD alpha chain and 145 kD beta chain forms of phospho-tyrosine (Y 1234/1235) cMET in 13 human breast tumor cell lines. The mature 45 KD form of cMET was detected primarily in SUM149 IBC cells as well as in AU565, MDA-MB-231, SUM159 and MDA-MB-468 breast tumor cells. The 145 kD beta chain form of phospho-Y 1234/1235 cMET was present in SUM149 cells, in SUM190, MDA-IBC3 and KPL-4 IBC cells and in AU565, MDA-MB 231, SUM159, MDA-MB-468 and in SKBR3 human breast tumor cells. In contrast, the 45 kD alpha chain form of phospho- Y 1234/1235 cMET was generally produced at low levels and detected in SUM149 IBC cells as well as in SUM159 breast tumor cells. (PDF 43 KB) [file 40064_2013_554_MOESM2_ESM.pdf]
